# 

**Published:** 1886-10

**Authors:** 


### CONTENTS.

ORIGINAL COMMUNICATIONS—

The Application of Intra-Uterine Pressure by the Tampon in Diseases of the Endometrium. By V. H. Taliaferro, M. D., Atlanta, Ga. .... 465

A Defence of Cocaine in Cataract Extractions in Answer to Dr. Calhoun's Article. By J. M. Hull, M. D., Augusta, Ga. .... 477

Earache. By W. Cheatham, M. D., Louisville, Ky. .... 481

Lectures on Diseases of the Skin. By Henry Wile, M. D., Atlanta, Ga. .... 485

Phimosis with Exaggerated Symptoms. By T. M. Holmes, M. D., Rome, Ga. .... 493

CLINIC REPORTS—

A Case of Chronic Cirrhotic Kidney. By H. McHatton, Macon, Ga .... 495

SOCIETY REPORTS—

Chicago Medical Society .... 498

CORRESPONDENCE—

Our Philadelphia Letter .... 507

Our New York Letter .... 509

BOOK REVIEWS—

A Reference Handbook of Medical Sciences .... 512

Antiseptic Surgery—The Principles, Modes of Application and Results of the Lister Dressing .... 514

A System of Practical Medicine by American Authors .... 515

The Physicians' Leisure Library Series .... 516

Books Received .... 517

EDITORIAL—

The International Medical Congress .... 518

Relief for the Medical Profession of Charleston .... 519

Medical Flag over Alcoholic Mixtures .... 520

Items .... 521

Our Advertisers .... 528

